# The Effect of Carbon Credits on Savanna Land Management and Priorities for Biodiversity Conservation

**DOI:** 10.1371/journal.pone.0023843

**Published:** 2011-09-14

**Authors:** Lucinda L. Douglass, Hugh P. Possingham, Josie Carwardine, Carissa J. Klein, Stephen H. Roxburgh, Jeremy Russell-Smith, Kerrie A. Wilson

**Affiliations:** 1 The Australian Government National Environmental Research Program, The Australian Research Council Centre of Excellence for Environmental Decisions and The University of Queensland School of Biological Sciences, St. Lucia, Queensland, Australia; 2 The Commonwealth Scientific and Industrial Research Organisation Ecosystem Sciences, Dutton Park, Queensland, Australia; 3 The Commonwealth Scientific and Industrial Research Organisation Sustainable Agriculture Flagship & The Commonwealth Scientific and Industrial Research Organisation Ecosystem Sciences, Canberra, Australian Capital Territory, Australia; 4 Research Institute for the Environment & Livelihoods, Charles Darwin University, Darwin, Northern Territory, Bushfires Northern Territory, Northern Territory Government, Winnellie, Northern Territory, Australia; University of Western Australia, Zimbabwe

## Abstract

Carbon finance offers the potential to change land management and conservation planning priorities. We develop a novel approach to planning for improved land management to conserve biodiversity while utilizing potential revenue from carbon biosequestration. We apply our approach in northern Australia's tropical savanna, a region of global significance for biodiversity and carbon storage, both of which are threatened by current fire and grazing regimes. Our approach aims to identify priority locations for protecting species and vegetation communities by retaining existing vegetation and managing fire and grazing regimes at a minimum cost. We explore the impact of accounting for potential carbon revenue (using a carbon price of US$14 per tonne of carbon dioxide equivalent) on priority areas for conservation and the impact of explicitly protecting carbon stocks in addition to biodiversity. Our results show that improved management can potentially raise approximately US$5 per hectare per year in carbon revenue and prevent the release of 1–2 billion tonnes of carbon dioxide equivalent over approximately 90 years. This revenue could be used to reduce the costs of improved land management by three quarters or double the number of biodiversity targets achieved and meet carbon storage targets for the same cost. These results are based on generalised cost and carbon data; more comprehensive applications will rely on fine scale, site-specific data and a supportive policy environment. Our research illustrates that the duel objective of conserving biodiversity and reducing the release of greenhouse gases offers important opportunities for cost-effective land management investments.

## Introduction

Investment in habitat protection and sustainable land management is critical in preventing further loss and decline of biodiversity, given unprecedented rates of species extinction and environmental degradation [1], [2], [3]. Since biodiversity conservation is constrained by limited resources and must compete with other societal priorities, investments in conservation must be as efficient and effective as possible [4], [5]. Emerging markets for ecosystem services such as carbon storage are potential sources for increased conservation funding and for promoting land use change that may benefit biodiversity.

A carbon market includes financial incentives for altering the management or use of land either to reduce green house gas emissions or increase carbon biosequestration and storage [6]. A carbon credit is measured as 1 tonne of carbon dioxide or equivalent greenhouse gas, CO_2_-e (gases differ in their global warming potential). Organisations or individuals may reduce their carbon footprint by purchasing carbon credits within a voluntary carbon market or a regulated emissions trading scheme. Targeted reduction of carbon emissions through avoided deforestation has been shown to benefit biodiversity even at a low carbon price of US$2–16 per tonne of CO_2_-e [7]. However many landscapes, such as tropical savannas, have large effects on carbon fluxes despite low levels of land clearing. These fluxes are caused by changes in fire and grazing regimes and habitat modification [8]. The potential biodiversity benefits resulting from managing existing habitat to increase carbon stocks in landscapes such as savannas remains largely untested.

In the extensive and globally significant savannas of northern Australia, two key processes both threaten biodiversity and release carbon: fires of high frequency and intensity and ecologically inappropriate cattle grazing regimes [9], [10], [11], [12], [13], [14], [15]. Historical fine-scale mosaic fire patterns resulting from traditional Aboriginal land management have been replaced by more wide-spread and intense wildfires that occur predominately late in the dry season [16], [17], [18]. This shift to homogenously-burnt landscapes threatens fire-sensitive species and habitat types especially in the high rainfall savannas [14], [16], [19], [20]. Inappropriate stocking densities, especially in the semi-arid, low rainfall savannas threaten biodiversity by modifying habitat structure [21], [22], [23], [24], [25], causing soil degradation, sedimentation and altering catchment dynamics [26]. Many introduced grasses for cattle, such as Gamba grass (*Andropogon gayanus*), are invasive and increase the intensity of fires [27], [28].

The impact of fire and grazing on savanna ecosystems and the carbon cycle interact in complex non-linear ways (see [29], [30]). Land degradation from livestock grazing reduces biomass accumulation and causes soil damage, consequently releasing stored carbon and decreasing carbon sequestration capacity [31], [32], [33], [34]. Reducing both fire frequency and the extent of dry season fires increases landscape carbon storage [35], [36], [37], [38], [39], [40] and is considered a priority to abate the Northern Territory's emissions of greenhouse gases [41], [42]. Grazing can reduce fuel loads and consequently prevent fire ignition through consumption and compaction. In the absence of fire, woody thickening can occur [32], [43], which may increase carbon storage through increased biomass [44], [45]. However, woody thickening also decreases pastoral productivity and consequently woody vegetation is often cleared or burnt, releasing greenhouse gasses [46]. Furthermore, intensive grazing reduces below ground carbon [34], [47] and the combined impact of fire and grazing can reduce tree density [48].

The two actions that are required to reverse land degradation and the associated loss of biodiversity values and increase carbon stocks are (1) fire management involving the ignition of low intensity fires early in the dry season to reduce fuel load continuity and decrease the potential for late dry season fires [39], [49] and (2) reduced stocking densities [32], [50]. The strategic manipulation of fire regimes can reduce fuel loads, benefit biodiversity, reduce emissions from wildfires and increase landscape carbon storage [35], [36], [37], [39], [49], [51]. Implementing sustainable stocking rates, rotational grazing and seasonal use grazing practices to improve soil management within Australia's rangelands is predicted to have large benefits to carbon [29], [52] and biodiversity [53]. Given the close relationship between land management activities and the carbon storage potential of savannas, an emerging carbon market presents a potentially important opportunity to increase the funding available for improved land management [54].

Our research explores a novel approach for identifying priority areas for the efficient allocation of improved land management for biodiversity conservation while accounting for potential carbon revenue generated from increased biosequestration. Our approach enables answering the following questions: (i) where are the priority locations for implementing improved land management targeted at biodiversity conservation? (ii) how do these locations and costs change when the potential revenue generated by carbon sequestration is deducted from the costs of conservation action? and, (iii) what are the opportunities and costs of meeting targets for both biodiversity and carbon simultaneously? We demonstrate our approach using a case study within the tropical savannas of northern Australia.

## Methods

**Figure 1:**
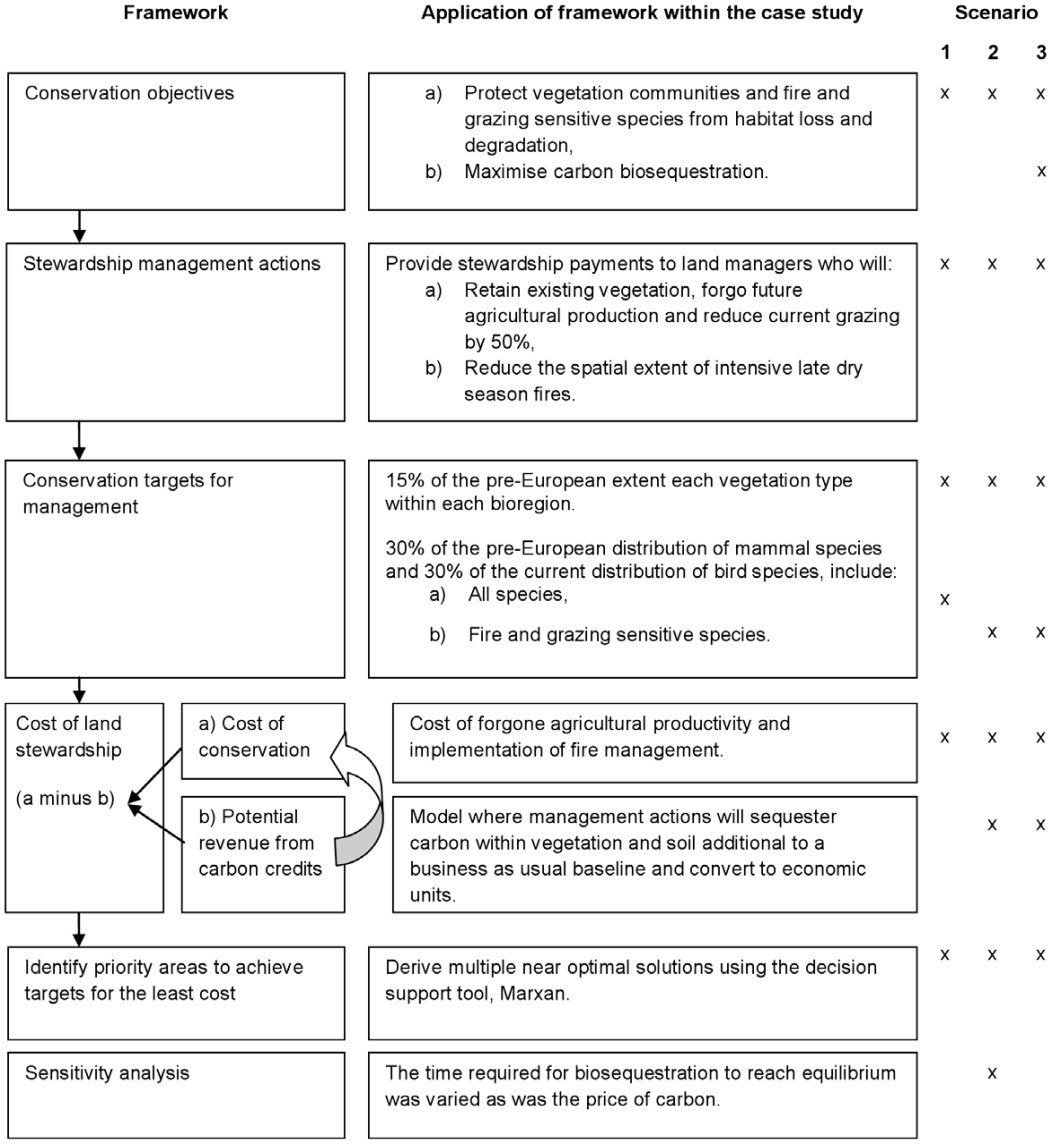
A framework to prioritise stewardship payments for improved land management while accounting for potential carbon revenue. Application of the framework to three scenarios in the context of Australia's northern savannas is described.

Contemporary conservation planning approaches follow five key steps: identifying objectives, actions, targets, costs and priority areas for implementation of conservation investment ([55], Figure 1). In Figure 1, we illustrate how we implemented each of these steps to identify land management priorities for improved grazing and fire management in Northern Australia. We used sub-catchments (n = 2,883) as our planning units, each which could be selected as a priority for land management [56]. For each planning unit, we summarized data on their biodiversity, conservation costs, and potential for carbon storage and revenue, described below.

### Biodiversity features

We represent the study area's biodiversity using the best available spatial data on vegetation types, bird species and mammal species of national environmental significance (refer to [57] for more detail). Vegetation types were derived using the National Vegetation Information System's major vegetation sub groups estimated prior to 1750 [58]. Heavily modified or cleared areas were removed using data from the Integrated Vegetation Cover [59]. Vegetation sub-groups within each Interim Biogeographic Regionalisation of Australia were treated as unique features. To represent the birds, we used distributions data that were modelled from point locality sightings between 1985 and 2005 [60], [61]. Species classed as an ‘incidental’ occurrence, introduced, vagrant, or wintering were removed. Sea birds and sightings without a date or grid reference were also excluded. Threatened mammal species as listed under the Environmental Protection and Biodiversity Conservation Act 1999 were collated from the Species of National Environmental Significance database [62]. Heavily modified or cleared areas were removed from the all species distribution data. We identified the bird species and threatened mammals that are sensitive to fire and/or grazing from the literature [21], [63]. In total, we considered 145 native vegetation types, 282 bird species, and 177 threatened mammal species.

### Conservation costs

For each planning unit, we estimated its conservation costs in terms of lost opportunity costs and fire management costs. We used stewardship costs derived by Carwardine et al. [57] to provide an estimate of payments required to compensate landowners for forgone agricultural profits from reducing pastoral income. The stewardship estimate was calculated using the most recent (1992–1997) agricultural profitability estimates at 1 km^2^ [64], which we adjusted for inflation up to 2006, a year representative of a comparatively stable global economy. As per Carwardine et al. [62], we assumed that reducing cattle grazing density by 50% would deliver biodiversity conservation outcomes and that landowners would accept a stewardship payment equivalent to forgone agricultural profits as compensation for reducing stocking rates and forgoing future grazing opportunities or any other opportunity resulting in habitat loss. The stewardship payment for each sub-catchment was calculated by multiplying the average profitability of the sub-catchments by the proportion of forgone agricultural profits (50%) and the area of native vegetation within the sub-catchment. The average annual lost opportunity cost for the study area was US$0.44ha^−1^.

The annual cost of fire management (2008–2009) was obtained for six Northern Territory Government Bushfire Regions (S. Sutton, unpublished data). We assumed that the cost of fire management would be greatest closer to more densely settled areas and human infrastructure due to the increased cost associated with safety precautions and management effort required to protect human life and property, but that it would also increase with distance from helicopter storage locations. The median remoteness of each Northern Territory Government Bushfire Region was calculated [58], [65] and plotted against the total cost of fire management within that region. We assigned a preliminary annual fire management cost to each remoteness category using the observed relationship and a minimum cost of US$0.025 ha^−1^ [49]. To allow for the helicopter travel and hover time to execute fire management activities, we assumed that fire management within 200 km of a helicopter storage location would be cheaper due to reduced necessity to refuel [66]. The preliminary fire management cost was multiplied by 1.5 for sub-catchments outside a 200 km radius from a helicopter storage location. The average annual fire management cost was US$0.06 ha^−1^.

### Carbon storage and revenue

We modelled the change in terrestrial carbon stocks resulting from improved land management. The predicted increase in carbon store above a business as usual baseline was converted to economic revenue and deducted from the cost of land stewardship. The variation in carbon storage under different scenarios was predicted using the carbon model, ‘AuSavan’. ‘AuSavan’ was designed for Australia's tropical savannas to evaluate carbon fluxes due to grazing, fire and drought and uses similar state and transition models as the Range-ASSESS model [67], [68], [69]. The major driving data of the model is 113 years of annual rainfall, annual rangeland growth, fire incidence, fire timing and stocking rates. A cycle of the AuSavan model therefore consists of 113 years. The default model parameter settings of Hill et al. [67] were applied with the following modifications to the grazing, prescribed fire and fire timing parameters.

We initially ran the model to generate business as usual baseline landscape carbon storage values. The cattle stocking parameter was set to 100% of current, as at 1997, levels [70]. No prescribed fires were introduced and the default late dry season fire thresholds were applied. These thresholds were used in the definition of the vegetation state transition rules to determine if a fire is considered ‘late season’, and therefore potentially more destructive [14], [67]. Under this simulation, the derived percentage of the burn area burnt in the late dry season (August–December) was 27%. We then reran the model to generate landscape carbon storage values under simulated improved management. Grazing was reduced by 50%, prescribed fires were introduced to all tenure classes, and the late dry season fire timing threshold was altered to minimise the occurrence of late season, and more intense fires [14]. This decreased the proportion of the burn area that was burnt by late season fires by 13%. The total burn area remained constant at approximately 50% of the study area.

Within the two aforementioned simulations, the long-term average, steady-state, total landscape carbon density was calculated and extrapolated to some areas not covered by the carbon model. The terms ‘steady-state’ and ‘equilibrium’ in reference to biosequestration refer to the state at which carbon stocks within the biosphere are fluctuating around a mean value over time with no net increase or decrease and are used here as a measure of the maximum achievable goal of a land-based carbon sequestration project. Following a similar methodology to Hill et al. [67], [69] we accounted for the variation in climatic, grazing and fire events by running the model for three cycles of 113 years for each Scenario to allow outputs to stabilise. The long term average, steady-state soil and biomass carbon values were then generated from a fourth cycle and summed to provide the total terrestrial carbon store. Two vegetation zones, rainforest and ‘other bush and shrub land’, are not accounted for in the carbon model. The carbon data from neighbouring cells were interpolated to these areas with the exception of 270 sub-catchments with less than 50% data coverage, which were excluded from the analysis.

### Combining conservation costs and carbon revenue

**Figure 2:**
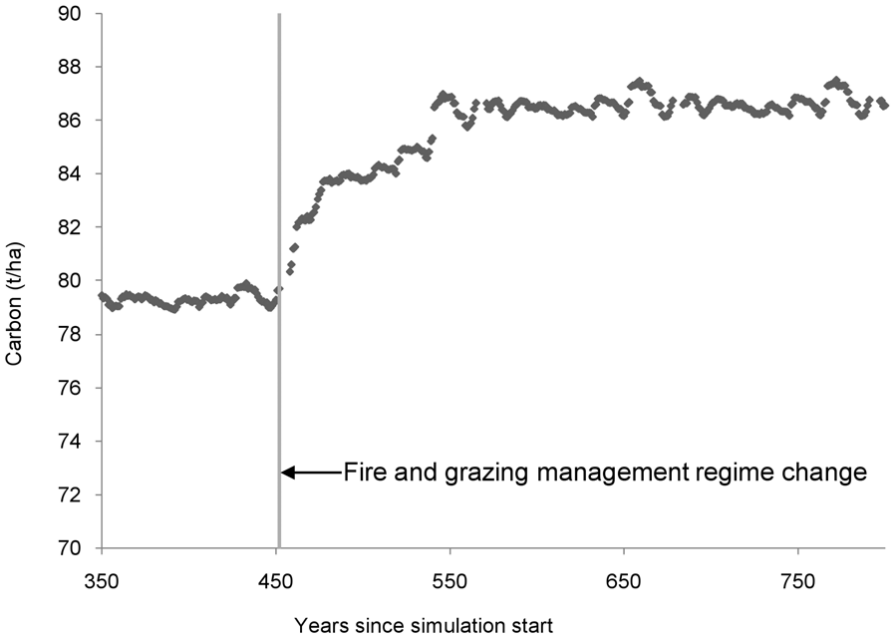
Carbon stock increases with a simulated improved fire and grazing management regime. The carbon density averaged across all vegetation zones was modelled by the state and transition carbon model, AuSavan [67]. The simulation was initiated using parameters representing a ‘business as usual’ scenario (i.e. no improved land management) and run for 4 cycles of 113 years ending at year 452. We then introduced a regime change by altering the parameters to reflect improved fire and grazing management.

The average annual carbon storage benefit was calculated, converted to revenue and deducted from the cost of conservation. We determined the amount of carbon that could be sequestered and retained, through improved land management by deducting the baseline carbon storage values from the carbon values under simulated improved land management (Figure 2). The carbon values were divided by the time required to reach a steady state to determine an average annual carbon storage benefit. The time taken for an ecosystem to reach a state of carbon equilibrium after improved management is implemented is uncertain, difficult to estimate and dependant on many factors [71]. We assumed two time periods, 20 and 90 years to test the sensitivity of the results to this uncertainty. The carbon density values were converted to carbon dioxide equivalents (CO_2_-e) by multiplying them by 3.66: the atomic mass of carbon dioxide divided by the atomic mass of carbon [72]. The carbon dioxide equivalents were multiplied by the price of one tCO_2_-e (varied from US$4 - 70 tCO_2_-e^−1^).

We combined and endowed all annual costs over 20 years. We assumed a discount rate of 5.7% per annum which is equivalent to the average Australian government bond rate between March 1999 and March 2009 for bonds maturing ten years after their initial issue. Inflation was assumed to be 3.1% per annum which is the average for the same time period. Similar to Carwardine et al. [57], a one-off transaction fee of US$7 ha^−1^ was added to each of the two conservation costs. Thus, the total cost of land stewardship within sub-catchment *i* is:
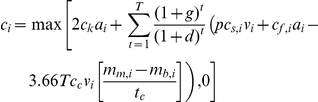
Where *c*_i_ is the total cost of land stewardship in each sub-catchment *i*, *c_k_* is the transaction fee, α*_i_* is area of sub-catchment *i*, *T* is the number of years of stewardship arrangement, *t* is the year of stewardship arrangement, g is the rate of inflation, *d* is the discount rate, *p* is the proportion of profit loss due to stewardship arrangement (set to 50%), *c_s_*_,*i*_ is the average annual cost of forgone agricultural profits from reduced grazing in sub-catchment *i*, *v_i_* is the area of vegetation in sub-catchment *i*, *c_f_*_,*i*_ is the average annual cost of fire management in sub-catchment *i*, *c_c_* is the price of 1 tonne of carbon dioxide equivalents, *m_m_*_,*i*_ is the average total carbon density after improved land management has been implemented in sub-catchment *i*, *m_b_*_, *i*_ is the average total carbon density under the business as usual Scenario in sub-catchment *i*, and *t_c_* is the time required for carbon to reach a new steady state post the implementation of land management. If *c_i_* is negative (that is, the net value of carbon benefits is greater than the net present value of the conservation costs) we set the total cost to zero.

### Identifying priorities

We used the decision-support software, Marxan [73], to determine priority sub-catchments for the implementation of improved management that would meet pre-specified conservation targets in a cost-effective manner. Marxan aims to achieve conservation targets (e.g. such as protecting a proportion of each habitat type) while minimising costs. Marxan was chosen over other reserve selection and optimisation algorithms since it seeks cost effective solutions and the simulated annealing algorithm can identify multiple solutions and analyse a large number of variably shaped planning units quickly [74], [75], [76].

Three land management scenarios were explored (Figure 1). For all scenarios, we set our baseline targets such that 15% of each vegetation type within each bioregion and 30% of the distribution of each fauna species should receive improved land management. These targets are based on Australian government forest conservation policies which state that 15% of each pre-European extent of each forest type or 30% of each ecological community should be minimum protection goals [77]. We then varied the targets between 25%, 50%, 75%, 100%, 125%, 150%, 175% and 200% of the baseline targets to test the sensitivity of the analysis to the target chosen and explore solutions above and below the baseline. Scenario 1 was our baseline scenario for comparison and did not consider carbon revenue or grazing and fire sensitive species (Figure 1). In Scenarios 2 and 3, we focussed on grazing and fire-sensitive bird and mammal species and factored in the potential revenue from carbon finance. In Scenario 3 we added a target for carbon so that the solutions met both biodiversity and carbon goals simultaneously. Carbon targets for Scenario 3 were based on the maximum amount of carbon that could be gained without compromising any biodiversity targets or increasing the overall cost (as determined by scenario 2) at each biodiversity target level. IUCN (International Union for Conservation of Nature) class I–IV reserves were forcibly included in all solutions.

We compared the selection frequency (i.e. the number of times the planning unit was selected across different solutions to the same problem) of priorities identified in scenarios 1 and 2 to determine whether incorporating carbon revenue would alter our conservation priorities. We also compared the net cost of the best solution of each scenario at each biodiversity target level.

Using the Bray-Curtis method, we determined the relative dissimilarity of 10 solutions between scenarios and within scenarios [78]. Subsequently, a hierarchical cluster analysis with complete linkage was performed and a dendrogram illustrating dissimilarity was created. The dendrogram was partitioned into clusters and an ordination using Kruskal's non-metric multidimensional scaling was used to plot solutions in two dimensional space [78]. A cluster analysis was performed to compare all scenarios at the baseline targets (with Scenarios 2 and 3 incorporating carbon at a price of US$14 CO_2_-e^−1^, and Scenario 3 targeting carbon at its maximum level for the fixed cost of Scenario 2) and for all scenarios across three different carbon prices (US$4, US$14, and US$70 tCO_2_-e^−1^).

## Results

The average combined soil and biomass carbon density of the study area increased with a simulated improved management regime consisting of a 13% reduction in the area burnt by late dry season fires and a 50% reduction in stock density (Figure 2). Carbon density fluctuated around 79 tC ha^−1^ when carbon model (AuSavan) parameters were set to reflect a ‘business-as-usual’ system. After the modifications to the land management regimes were introduced the carbon density increased rapidly, then more gradually, over 90 years of simulation before equilibrating at approximately 86 tC ha^−1^. The difference between carbon at equilibrium, before and after the introduction of improved management, was approximately 7 tC ha^−1^ when calculated by AuSavan as an average across all vegetation zones (Figure 2) and 11 tC ha^−1^ when refined to our study area as per the data processing methods described above in the carbon storage section (i.e. after extrapolating data to shrubland sub-catchments then averaging carbon values within sub-catchments). The resulting average annual sequestration rates were 0.55 tC ha^−1^ yr^−1^ and 0.12 tC ha^−1^ yr^−1^ for the two assumed sequestration periods, 20 and 90 years, respectively. The annual carbon revenue generated was US$5 ha^−1^ yr^−1^.

When biosequestration was included at a price of US$14 tCO_2_-e^−1^and a 90 year time period was assumed to achieve equilibrium, the costs of land stewardship were reduced by 76%. The proportion of sub-catchments for which the cost could potentially be reduced by carbon revenue was 71%. The average cost of the stewardship arrangement was reduced to US$5 ha^−1^ and for 68% of sub-catchments the stewardship cost was reduced to zero.

**Figure 3:**
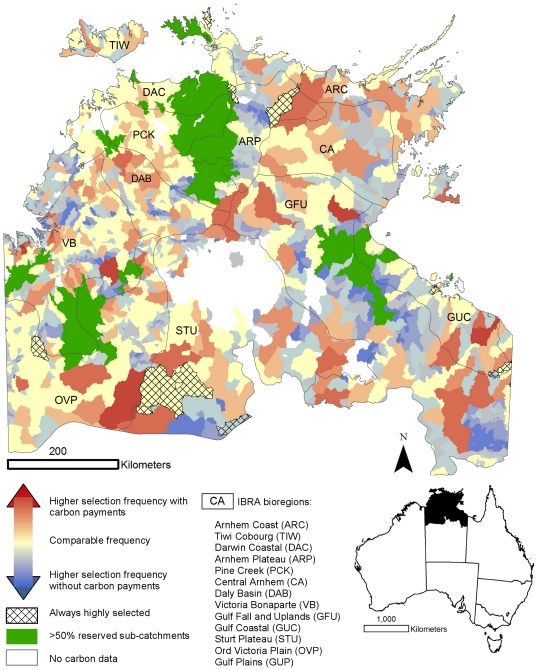
A comparison of the difference in selection frequency, a measure of investment priority, between scenarios 1 and 2.

The mapped selection frequency of each sub-catchment for Scenarios 1 (biodiversity only) and 2 (biodiversity and potential for carbon revenue considered) shows different sub-catchments being selected (Figure 3). Sub-catchments more frequently selected in Scenario 1 (blue sub-catchments) include those within the central Gulf Falls and Uplands, southern Darwin Coastal and central Arnhem Plateau regions. When carbon revenue is included (Scenario 2) some sub-catchments became more frequently selected (red sub-catchments). There was some similarity between scenarios with a number of sub-catchments selected more than 80% of the time in both Scenarios 1 and 2.

**Figure 4:**
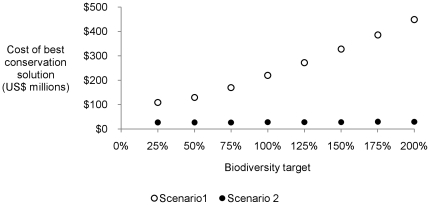
The least cost conservation solution generated by Marxan for Scenario 1 at a carbon price of US$14 tCO_2_-e increased steadily as the biodiversity target level was increased from 25% to 200% of the baseline targets. The cost for Scenario 2 (where carbon revenue is deducted from the cost of land stewardship) remained comparatively constant as the carbon captured increases with the larger biodiversity targets (Figure 5).

**Figure 5:**
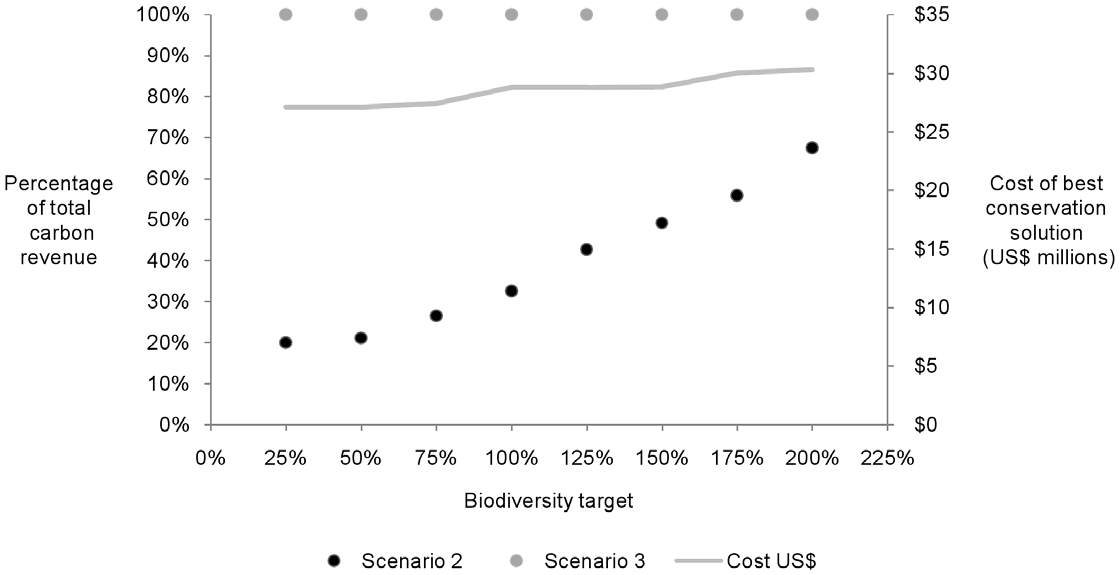
The total carbon potential of the stewardship arrangement was achieved for all biodiversity target levels when carbon biosequestration (tCO_2_-e) was specifically targeted (Scenario 3) and when a budget was fixed equivalent to the cost of Scenario 2 (where carbon revenue is deducted from the land stewardship cost) at the same biodiversity target level. The amount of incidental carbon captured within Scenario 2 rose with an increase of the biodiversity target and the cost.

The cost of the best conservation solution for Scenario 1 increased with an enhanced biodiversity target level from approximately US$100 million at a low target (25% of baseline) to US$200 million (100% of baseline) and US$450 million (at 200% of the baseline target; Figure 4). For Scenario 2, the cost of the best solution was much lower; around US$27 million and remained comparatively steady with increased targets. While more area is required to meet the increased biodiversity targets, more carbon is able to be sequestered in this area (incidentally in the case of Scenario 2; Figure 5) and therefore potentially more credits can be obtained to offset the costs of land stewardship. The difference in cost between Scenarios 1 and 2 is approximately US$73 million at the 25% biodiversity target level and US$420 million at the 200% target. This is a saving of 73% to 93% respectively.

The total potential of the study area for biosequestration through improved fire and grazing management was prioritised when biosequestration was specifically targeted (Scenario 3) across all biodiversity target levels. Under this Scenario, the increase in cost between the lowest biodiversity target level (25%) and the highest (200%) was approximately US$3 million (Figure 5).

The Bray-Curtis analysis of dissimilarity of solutions showed that solutions for all scenarios formed distinct clusters indicating that solutions between scenarios are more dissimilar than solutions within scenarios. Solutions for Scenarios 1 and 2 have a comparative dissimilarity of 10%. Scenario 3 solutions were 55% dissimilar to those of Scenarios 1 and 2.

Ordination (using Kruskal's non-metric multidimensional scaling) of solutions showed that Scenario 2 solutions across two carbon prices (US$14 and US$70) for each of the two time periods assumed for carbon to reach a steady state (20 and 90 years) formed no distinct clusters. This trend was also observed for the carbon price US$4 and 20 year time period. Dissimilar clusters were identified when the cost was calculated for the 90 year time period and for a low carbon price (US$4).

## Discussion

We provide an analysis framework capable of prioritising stewardship payments for improved land management to achieve biodiversity conservation goals within the context of an emerging carbon economy. Rather than opting for a standard conservation planning approach focussing on the creation of new protected conservation reserves [55], [79], we explicitly account for the utility that land stewardship programs offer for biodiversity conservation. Our analysis shows that priorities for land stewardship payments that account for an emerging carbon market are likely to deliver greater benefits at a lower cost than if the carbon market is ignored. These results have important implications for policy and planning.

We show that by reducing both the area burnt under late season fires by 13% and stocking density by 50%, we increase the study region's potential for carbon storage by 350–600 million tC or 7–11 tC ha^−1^, after approximately 90 years. This carbon benefit equates to an atmospheric saving of between 1–2 billion tCO_2_-e which would offset Australia's agricultural emissions [80], for the next 22 years. Furthermore, this biosequestration potential is only a fraction of the potential for emissions reductions from Australia's land use sectors which is expected to be approximately 1BtCO_2_yr^−1^ for 20–50 years [52].

Our results reflect previous predictions from carbon sequestration studies in northern Australia. Murphy et al. [37] estimated that fire management alone could increase woody biomass carbon stocks by 6.1 tC ha^−1^ over the next century in fire-prone regions, which is equivalent to a carbon offset of 22 tCO_2_-e ha^−1^. The higher values found in our study region could be attributed to the addition of grazing management and accounting for soil carbon. Other recent studies have suggested carbon sequestration rates from fire management ranging from 0.5 tC ha^−1^ yr^−1^ [35] to 0.7 tC ha^−1^ yr^−1^ [36] but do not suggest an upper limit to this potential. Harms and Dalal [81] report an average 9.7% decrease in the carbon content of grazed soils when comparing sites that have been heavily grazed over a long time period with those that are relatively undisturbed. This is comparable to our observed 8% increase in overall carbon density for a region with a short history of lower intensity grazing while accounting for biomass carbon and improved fire management, in addition to soil carbon.

Previous research shows a range of biosequestration responses to changed grazing and fire regimes, some of which are much lower and much higher than that observed in this study [82]. This diversity of responses has been attributed to the variation in climate, microbial community, nutrient cycles, litter chemical composition, and the pre-existing management regime between experimental sites. Future climate change is generally predicted to worsen fire weather; the weather variables that influence fire behaviour, ignition and suppression [83]. Predictions of future fire weather within northern Australia are inconsistent [84]. Site-specific carbon data is urgently required, in addition to an improved understanding of future variation of, and the synergies between, fire, grazing, and the carbon cycle as further discussed below.

We find that by accounting for the potential for economic returns from carbon revenue through improved land management, the priority sub-catchments for conservation investment changes (Figure 3). Carbon revenue from improved fire management and reduced stock density diminish the cost of conservation (Figure 4) in over 70% of the sub-catchments and completely offset the cost of implementing improved land stewardship in almost all of these. This increase in cost competitiveness between sub-catchments influenced the priority sub-catchments for conservation investment. There are however, some sub-catchments that were priorities for conservation investment irrespective of a carbon market (Figure 3). Sub-catchments of consistent priority include those within the Arnhem Plateau, Gulf Coastal, Ord Victoria Plain, Tiwi Cobourg and Sturt Plateau bioregions. These sub-catchments contain restricted range species such as the Carpentarian Rock-rat (*Zyzomys palatalis*) and are also cost-effective.

Our analysis indicates that it is possible to conserve more biodiversity and maintain carbon stocks for the same budget by explicitly seeking to achieve both objectives. The addition of carbon revenue has the potential to increase the budget available for biodiversity conservation, which increases the number of sub-catchments that can be actively managed. Similarly, this approach can reduce the cost of achieving conservation goals by accounting for the potential for carbon revenue to offset the costs of land stewardship. Our research shows that we can capture the total biosequestration potential (i.e. the total amount of carbon that can be sequestered and retained through the specified improved land management regime) without sacrificing the achievement of biodiversity targets by specifically targeting biosequestration as a conservation goal (Scenario 3, Figure 5). This is an increase of 35–80% above the amount of carbon incidentally captured under a conservation focussed plan (Scenario 2) and illustrates the value of setting specific objectives for all goals simultaneously. We acknowledge that biodiversity conservation and biosequestration objectives are not always mutually beneficial [85], [86] and appropriate policy and careful planning is required to prevent perverse outcomes.

In order to improve the accuracy of this analysis, expenses associated with carbon verification (that is, the cost of demonstrating that management has led to a measurable change in ecosystem carbon storage) would need to be refined. We partially account for carbon verification cost by incorporating a transaction fee and including a minimum cost for fire management derived from data, but ideally the cost of field surveys and remote sensing analyses should be included.

Our results are constrained by the uncertainty and inaccuracies inherent in the carbon model employed in this analysis. The focus of this research is to provide and exemplify an analysis framework and therefore many carbon models would have sufficed to provide indicative but realistic data. The carbon model ‘AuSavan’ incorporates the many inter-relations required to determine the effects of grazing and fire on Northern Australia's savanna carbon stocks [67]. It is however reliant on the spatial resolution and quality of input data, much of which is over ten years old. AuSavan is based on 113 years of historic climate and fire data [67], [69], whether this reflects future trends such as changed fire regimes due to climate change [83], is untested. The baseline carbon values are derived from a model that estimates biomass and soil carbon values across Australia from a limited number of field observations (n = 76), with approximately one quarter of those located in the tropical regions of Australia [87]. Furthermore, the carbon values and transition rules are applied evenly across vegetation zones and do not account for variability of vegetation structure or soil dynamics that are affected by topography, microclimate, or the impacts of cyclones [37], [88]. Overall, the uncertainty associated with landscape scale carbon models is typically high due to natural heterogeneity across large landscapes. The models employed in this analysis have a coefficient of variation of carbon emissions of approximately 20–30% [87]. Further refinement and field calibration of the underlying carbon models, particularly the prediction of biomass carbon density, would help reduce the uncertainty. The value of improving data due to the logistical constraints of field sampling across large areas needs however to be weighted against the consequences of the uncertainty for environmental decision-making. In our analysis we found that the results were relatively insensitive to the time assumed for carbon to reach a new steady state. However, when this uncertainty is also combined with other uncertainties associated with an emerging carbon market (specifically the value of carbon), different conclusions might be drawn.

We find that for a moderately low and high carbon price of US$14–US$70 and a 20–90 year time period for carbon to reach equilibrium, the similarity of solutions was high, but this was not the case if the price for carbon was very low and the time to reach equilibrium was long (US$4 tCO_2_-e^−1^ and 90 years). The annual biosequestration benefit reduces with an increasing time to reach equilibrium. Therefore, the cumulative impact of a low carbon price and longer time period for biosequestration to reach equilibrium is a reduction in the overall economic benefit of carbon credits. We assume that biomass and soil biosequestration will attract the same cost. The global average carbon price across various trading schemes is around US$20 [89] and carbon transactions in Australia are projected to range between US$10 to US$52 within the next 30 years [90]. Furthermore, offsets that deliver complementary benefits such as biodiversity conservation are likely to command a premium [91].

We have used fire seasonality as a surrogate for fire intensity [14], [19]. The commonly accepted fire management paradigm supports the assumption that early season fires will benefit biodiversity by reducing the frequency of more intense late season fires [18]. Nevertheless, early season fires may negatively affect juvenile trees [92] and some species require intense fires for germination [93], [94]. Also, fire intensity is intra-seasonally heterogeneous and severe fires can occur early in the dry season [19]. There is no explicit ‘intensity’ aspect in the carbon model used in this analysis. AuSavan assumes that less of both fire and grazing will leave more fuel after grazing which increases the probability of fire incidence given favourable climatic conditions [67]. Fire intensity is manifest through the resulting state changes which are functions of fire timing, fire frequency and grazing (e.g. open woodland transiting to thinned woodland).

Further research and policy development is also required to clarify what land use, land use change and forestry activities will be accounted for under future international (e.g. post-Kyoto Protocol) and national compliance emissions trading schemes, how these will be measured and how they interact [54]. Recent research and commentary has provided a better understanding of the technical aspects of landscape carbon dynamics [29], [30], [35], [36], [39], [95], [96] and of the national and international policy environment [44], [49], [97]. The applicability of our approach depends on a favourable policy and economic environment as well as the concordance between land management methods that increase carbon storage and those required for biodiversity conservation.

The implications of our research are significant for biodiversity conservation and also for the livelihood outcomes that can be generated through carbon abatement and stewardship arrangements [49]. Our research is primarily focused on the issue of conserving biodiversity. However, there are also important social considerations that could be incorporated into this analysis such as the contribution of land management projects and a carbon economy to achieving livelihood objectives. Furthermore, northern Australia's savannas are considered of very low pastoral potential [98], are only partially grazed and rely heavily on public subsidies. Therefore, land management that generates carbon revenue, especially through fire management, may provide a viable and ecologically advantageous alternative [29].

In summary, we provide an analysis framework capable of prioritising locations for land management that improves biodiversity conservation while accounting for conservation costs and potential revenue from carbon finance. We find that improved fire and grazing management has the potential to deliver carbon revenue that can be used to offset the cost of land stewardship. Our preliminary results show that we can conserve more biodiversity, capture more carbon and reduce the cost of conservation if we explicitly integrate each of these aspects in the development of conservation plans. By prioritising investment in stewardship programs, we account for the flexibility and utility offered by community partnerships to implement conservation actions within remote and expansive regions, such as Australia's tropical savannas [97], [99].
